# Iron-Sulfur Clusters: A Key Factor of Regulated Cell Death in Cancer

**DOI:** 10.1155/2022/7449941

**Published:** 2022-10-26

**Authors:** Mengning Zhang, Zhijun Liu, Yifei Le, Zhenqi Gu, Hong Zhao

**Affiliations:** ^1^The First School of Clinical Medicine, Zhejiang Chinese Medical University, Hangzhou, China; ^2^School of Life Science, Zhejiang Chinese Medical University, Hangzhou, China; ^3^The First Affiliated Hospital of Zhejiang Chinese Medical University (Zhejiang Provincial Hospital of Traditional Chinese Medicine), Hangzhou, China

## Abstract

Iron-sulfur clusters are ancient cofactors that play crucial roles in myriad cellular functions. Recent studies have shown that iron-sulfur clusters are closely related to the mechanisms of multiple cell death modalities. In addition, numerous previous studies have demonstrated that iron-sulfur clusters play an important role in the development and treatment of cancer. This review first summarizes the close association of iron-sulfur clusters with cell death modalities such as ferroptosis, cuprotosis, PANoptosis, and apoptosis and their potential role in cancer activation and drug resistance. This review hopes to generate new cancer therapy ideas and overcome drug resistance by modulating iron-sulfur clusters.

## 1. Introduction

Iron-sulfur clusters are ancient, omnipresent cofactors composed of iron and inorganic sulfur and are involved in many important biological processes. Around 2.4 billion years ago, before the Great Oxygenation Event, the early earth was in a low-oxygen environment and was rich in reducing Fe and S [1]. As the iron-sulfur clusters are made up of the primitive yet abundant reducing elements, they were believed to be readily available prosthetic groups that life exploited early on during evolution and spontaneously assembled into primitive biological macromolecules by using suitable ligands [2, 3]. As iron-sulfur cluster proteins formed in an anaerobic atmosphere, they oxidized iron to its insoluble Fe^3+^ state and produced molecular oxygen that led to photosynthesis [4]. As a result of such unfavorable conditions, biogenesis systems developed iron-sulfur clusters to protect them from high oxygen concentrations that could damage DNA [5]. Consequently, iron-sulfur clusters play an essential role in enzymes involved in oxidation-reduction reactions, DNA synthesis and repair, tRNA modification, and a variety of other cellular functions [6, 7]. A recent study highlighted that iron-sulfur clusters served as cofactors for the SARS-CoV-2 RNA-dependent RNA polymerase and were assigned as targets for the COVID-19 therapy [8].

With the growing evidence that cancer cells preferentially depend on iron relative to normal cells [9], Fe–S biogenesis can somewhat be theorized to represent a fundamental difference between cancer and nonmalignant cells. However, the underlying rationale remains elusive. As for the critical role that Fe–S metabolism plays in cancer development, progression, and therapeutic resistance, the modulation of iron-sulfur clusters may become a golden key in cancer therapy, which is heavily dependent on regulated cell death as a key determinant of success. It is proved that several genes involved in [2Fe-2S] type of iron-sulfur cluster synthesis and transport being overexpressed in multiple cancer types [10]. Further, iron-sulfur assembly scaffold (ISCU) is downregulated in cancer to improve cancer cell survival, while ISCU inhibition has led to worsened clinical prognosis in patients with breast, head, and neck cancer [11], suggesting the potential for modulating iron-sulfur clusters in the clinical treatment of cancer. Moreover, multiple novel types of cell death, such as ferroptosis, pyroptosis, PANoptosis, and apoptosis are proven to play a vital role in the depression of tumorigenesis by removing cancer cells [12–14]. Thus, this article reviews the significant role of iron-sulfur clusters in cancer cells and multiple types of novel nonapoptotic-regulated cell death. The findings of this study will provide new ideas of regulating iron-sulfur clusters to improve cancer treatment.

## 2. Ancient Iron-Sulfur Clusters: Bring Forth New Stories through the Old

### 2.1. Structure and Function of Iron-Sulfur Clusters

Iron-sulfur clusters were first discovered in the early 1960s through enzyme purification using characteristic electron paramagnetic resonance signals. Iron-sulfur proteins have been discovered in plant and bacterial oxygen-reducing proteins, as well as mitochondrial and bacterial respiratory complexes. Iron-sulfur cluster complexes are synthesized by these proteins, which act as catalysts, stabilize protein structures, and perform regulatory functions [15, 16], making them important for essential physiological pathways in living cells.

Iron-sulfur clusters are versatile cofactors in facilitating various chemical activities [7]. Among enzymes such as mammalian ferrochelatase, mitochondrial respiratory complexes I and II, ferredoxins (FDXs), and Rieske proteins, iron-sulfur clusters are most commonly rhombic [2Fe-2S] [17]. Ferrochelatase (FECH), the terminal enzyme of the heme synthesis pathway, contains a [2Fe-2S] cluster that is essential for its functional activities [18–20]. Another common example is the tetranuclear [4Fe-4S] cluster, which is capable of accepting or donating single electrons. Specifically, the catalytic subunit of DNA polymerase *ε* contains a [4Fe-4S] cluster [21]. The flexible iron-sulfur clusters can function as modules that reassemble into more complex structures [17]. A cubane cluster can be formed by reductive coupling of two [2Fe-2S] clusters [22]. Furthermore, [8Fe-7S] P-clusters or [7Fe-9S] iron-molybdenum cofactors have an interstitial carbon atom in their core, adding to their complexity [7].

Iron-sulfur clusters perform three biological functions: electron transfer, enzyme catalysis, and biological regulation [23]. The biological electron transport is mediated primarily by iron-sulfur clusters due to their delocalization of electron density over Fe and S atoms [24].

Since iron-sulfur clusters usually play a key role in enzymatic processes, the protein function largely depends on forming iron-sulfur clusters through mitochondrial biogenesis [25]. Heme synthesis and iron-sulfur biogenesis take place on the inner mitochondrial membrane [26]. Regulation of heme synthesis by ferrochelatase (FECH), the terminal enzyme of the heme synthesis pathway, requires the presence of a [2Fe-2S] cluster that is required for its functioning [20, 27].

The versatile chemical properties of iron-sulfur proteins have facilitated their widespread use in almost all organisms to perform many reactions involving basic cellular processes such as respiration, photosynthesis, metabolism, and nitrogen fixation. Among these proteins, cysteine desulfurases NFS1, ISU1, and ISU2 play a central role in iron-sulfur cluster assembly in mitochondria [28]. NFS1 proteins act as sulfur donors during the biogenesis of iron-sulfur proteins [29, 30]. They deliver sulfur to the ISU proteins, regarded as the scaffold proteins in forming iron-sulfur clusters [31]. Frataxin (FXN) is an essential iron-binding protein highly conserved in most organisms [32]. Studies have shown that reduced levels of FXN are responsible for causing the neurodegenerative disease of Friedreich's ataxia [33]. The deficiency of frataxins can also lead to various metabolic disturbances, such as oxidative stress, deficiency of iron-sulfur clusters, defects in heme synthesis, sulfur amino acid, and mitochondrial function. It has been shown that a highly conserved globular domain at the C-terminus of FXN is coupled to a flexible N-terminal region only found in eukaryotes [32]. FXNs directly bind to iron but have the very unusual property that their iron coordination is achieved only by exposure to glutamate and aspartate on the protein surface. In yeast and mammals, the interaction between proteins in the iron-sulfur cluster assembly is more distinct. In particular, NFS1, ISCU, ISD11, and FXN from the iron-sulfur cluster core complex [34–36]. The formation of the iron-sulfur cluster begins with a homodimer by NFS1 on which monomers of the scaffolding protein ISCU bind, near the top and bottom [37]. The cofactor pyridoxal 5′-phosphate facilitates the supply of inorganic sulfur from cysteine residues to NFS1, which then binds to the cysteine ligand provided by ISCU, and covalently binds to iron [38]. After that, the core complex recruits the NFS1-binding protein ISD11 and finally FXN [33, 39]. Ultimately, the iron-sulfur cluster is transferred to the receptor apolipoprotein via the binding of ISCU to the chaperone protein, forming a complete iron-sulfur cluster [40].

Regulatory proteins containing iron-sulfur cluster cofactors form an important group. They have a range of functions, including sensing molecular oxygen, stress responses, and iron regulation. These clusters play a central role in controlling the activities of the regulators as sensory modules. The clusters are required for the protein to reach its regulatory form in some cases, while in others, the clusters must be lost or modified for it to reach its active form [41]. In eukaryotes, iron-sulfur proteins play an important role in maintaining the genome, translating proteins, converting energy, and fighting infections [26]. Recent studies suggest that the differing demands for iron-sulfur biogenesis by tumor and nonmalignant cells underlie the demand for Fe-dependent cell growth [25]. Therefore, iron-sulfur cluster biogenesis is critical in exploring the roles of iron-sulfur clusters in cancer therapy.

### 2.2. Regulation of Iron-Sulfur Clusters

Although the significant increase in research on iron-sulfur clusters in recent years and the widespread interest in the role of iron-sulfur clusters in various diseases, including cancer, research on specific regulators of iron-sulfur clusters and their regulatory mechanisms is still very limited. Here, we summarize several substances that inhibit or agonize the assembly of iron-sulfur clusters, hoping that they may serve as a reference for targeting iron-sulfur clusters as a form of treatment for various diseases.

In physiological and stressful conditions, the iron-sulfur cluster assembly transcription factor (ISCR) acts as a sensor of cellular iron-sulfur levels and as a transcriptional regulator of iron-sulfur biogenesis [42, 43]. The RNA polymerase recognition sites contain two ISCR-binding sites that may prevent the RNA polymerase from binding to the promoters, resulting in repression of the iron-sulfur clusters operon expression [44]. The ISCR regulates iron-sulfur biogenesis under physiological and stress-induced conditions by manipulating the iron-sulfur clusters. In addition, as a result of its inactivation, the ISCR contributes to iron deficiency phenotypes [44].

In addition to biological regulators, small molecule compounds have been reported to directly regulate the assembly of iron-sulfur clusters. Factors required for the assembly of iron-sulfur clusters and apolipoprotein target maturation in *S. aureus* include Suf(sulfur-formation)S, SufBCD, SufU, SufT, SufA, and Nfu, where SufS provides sulfur from cysteine to SufU or SufBCD, which synthesizes [2Fe-2S] or [4Fe-4S] clusters [45]. Molecularly, VU0038882 (′882) inhibits the synthesis of iron-sulfur clusters by inhibiting the Suf complex, which synthesizes these clusters [46].

The anticancer effects of several anticancer drugs were shown to be at least partially dependent on the modulation of iron-sulfur clusters. *β*-Phenylethyl isothiocyanate (PEITC), a natural anticancer product highly effective against human leukemia, increases ROS production with depletion of the mitochondrial antioxidant glutathione, acting at least partially through increased ROS, leading to degradation of the iron-sulfur cluster center in NDUFS3 as part of ETC complex I [47]. MAD-28 is a derivative of cluvenone, a mitochondrial targeting molecule with anticancer properties and good tumor selectivity [48]. MAD-28 destabilizes the iron-sulfur clusters by breaking the coordinated bond between the histidine ligand and the iron of the mitochondrial NEET and NAF-1 iron-sulfur clusters [49]. As a result of this action, MAD-28 can strongly and with high specificity inhibit the proliferation of cancer cells [50]. The anthracycline doxorubicin is a widely used chemotherapy drug that causes cardiotoxicity and cardiomyopathy. Doxorubicin acts as an iron chelator in cardiomyocytes so that a complex is formed between iron and doxorubicin, catalyzing the conversion of hydrogen peroxide to hydroxyl radicals, associated with functional impairment of mitochondria, destruction of iron-sulfur clusters, and an increase in free iron in mitochondria [51]. Furthermore, several drugs and small molecule agents of natural product origin have also shown promising potential value for the regulation of iron-sulfur clusters in cancer therapy [52].

Although the important role played by iron-sulfur clusters in various biological processes and their mechanisms has been explored repeatedly, the regulation of these processes by modulating iron-sulfur clusters has been rarely reported. This may become a critical point for future research direction on iron-sulfur clusters.

## 3. Iron-Sulfur Clusters and Cancer

In recent years, there has been an increasing amount of research and widespread attention on iron-sulfur clusters and their important role in various diseases. Among these, the important role of iron itself in the cell cannot be ignored. Although iron is essential in living cells, its overabundance is harmful. The presence of excess iron may result in an increase in cellular oxidative stress. This can be achieved through the reaction of iron with dioxygen, and classical Fenton chemistry, which produces highly reactive hydroxyl-radical from H_2_O_2_ (HO•) [53], resulting in organic hydroperoxides, organic radicals, and the oxidation of aldehyde by-products of lipids and amino acids. There is therefore a link between altered iron metabolism and several diseases, including Alzheimer's disease, Parkinson's disease, and chronic kidney disease [54, 55].

Compared to nonmalignant cells, cancerous cells preferentially take up and sequester iron. Novel research also hypothesized that iron is a central link between genetic and metabolic cancer theories [56]. During the period 1997-2008, 309,443 Taiwanese adults without previous cancer histories had their serum iron levels tested. As a result of a link between the National Cancer Registry and the National Death File, iron levels were initially found to be associated with subsequent cancer risk. Following long-term follow-up, participants had a 25% increased cancer incidence risk and a 39% increased cancer mortality risk [57]. Mechanically, iron regulatory proteins are frequently altered in cancer cells [56]. Cancer cells can frequently upregulate transferrin as well as downregulate ferroportin, which increases the steady-state level of intracellular redox-active iron, which is known as the labile iron pool (LIP) [58]. There is typically 2% intracellular iron in the LIP, which is mainly ferrous iron (Fe^2+^), thus causing oxidative stress [59], contributing to increased DNA damage [60]. It is shown that LIP increased in several cancer cell types relative to adjacent normal tissue [61, 62]. LIP of lung and brain cancer cells may increase more than twofold relative to normal human cells [62]. It is likely that iron metabolism and LIP play a central role in linking the mutational theory of cancer to the metabolic theory of cancer, as increased LIP can lead to increased mitochondrial uptake of iron and genetic instability.

Fe is utilized intracellularly in three ways: Fe–S biogenesis, heme synthesis, and mono- and di-iron proteins [63]. Particularly, Fe–S metabolism plays a crucial role in maintaining genomic stability, since DNA polymerases and DNA helicases are among the DNA metabolic enzymes that require iron-sulfur clusters to function properly [5, 6, 64]. Since it is considered that genomic instability is one of the primary hallmarks of cancer and plays a key role in neoplastic transformation [65], iron complexes in functional proteins, iron trafficking, and forming these functional cofactors are essential in cancer progression.

### 3.1. Cancer Initiation

Cancer cells have shown a preference for iron intake and sequestration relative to nonmalignant cells [56]. The transferrin receptor (TfR) is frequently upregulated in cancer cells, suggesting that iron flux may be increased in cancer cells [66]. TfR expression is transcriptionally regulated by c-Myc (a transcription factor encoded by proto-oncogene c-myc) and hypoxia-inducible factor 1*α* (HIF-1*α*).

Hence, an association exists between expression of C-Myc and HIF-1*α* with tumor aggressiveness [67, 68]. On the contrary, cancer cells can promote the degradation of iron transporter protein (FPN-1) by producing iron-regulatory elements, thereby limiting iron export [69]. Therefore, iron-sulfur clusters in cancer initiation become a point of worthy exploration.

Iron-sulfur cluster biogenesis plays an integral role in cancer activation. Instabilities in the genome can be caused by defects in mitochondrial and cytoplasmic iron-sulfur cluster biogenesis, as well as the insertion of nuclear iron-requiring enzymes involved in DNA synthesis and repair. It was shown that several genes related to iron-sulfur cluster biogenesis were altered in tumor tissues compared to their normal counterparts, with several genes involved in [2Fe-2S] synthesis and transport being overexpressed in multiple cancer types [10]. The majority of [2Fe-2S] synthesis is upregulated, but ISCU may be downregulated. It may be related to the fact that ISCU is positively regulated by p53 through intron binding sites, and that it is reduced in human liver cancer tissues [70]. In addition, ISCU is downregulated in cancer to improve cancer cell survival, while ISCU inhibition has led to worsened clinical prognosis in patients with breast, head, and neck cancer [11]. FXN is a mitochondrial protein involved in iron-sulfur cluster biogenesis. In cancer cells, FXN overexpression is associated with hypoxia-induced tumor stress via the HIF pathway [71]. The expression of FXN is increased in a variety of tumor cell lines in response to hypoxic stress, which is often associated with the progression of cancer. Furthermore, a hypoxia-induced increase in frataxin is dependent on hypoxia-inducible factors and regulates the activation of p53 [72]. Hypoxia-induced stress in tumors is mediated by FXN, which may contribute to tumor cell survival and progression. Furthermore, as mentioned before, reduced levels of FXN are responsible for causing the neurodegenerative disease of Friedreich's ataxia [33]. One case study had reported the development of gastric and breast cancers in siblings with Friedreich's ataxia, suggesting that Friedreich's ataxia is also related to cancer [73].

Existing studies suggest that since the metabolic network of iron-sulfur clusters plays a critical role in cancer [74–76], compounds currently under clinical investigation targeting complex networks of iron-sulfur metabolism may provide a novel approach to inhibit cancer progression by selectively disrupting cancer cell metabolism by exploiting differences between cancer and nonmalignant cells. Available studies suggest several possible ways to disrupt the metabolic network of iron-sulfur clusters on different levels [25]. First, redox operations can be used to destabilize iron-sulfur clusters. Second, increasing iron chelation can limit cellular iron availability to prevent cluster formation. Finally, replacing Fe with redox inert metals can prevent iron-sulfur biogenesis and limit the function of iron-sulfur-containing proteins. These methods provide potential therapeutic strategies to target iron-sulfur clusters during cancer initiation.

### 3.2. Drug Resistance

Drug resistance remains a major limiting factor in obtaining a cure for cancer patients [77]. Although the initial success of early chemotherapeutic agents such as nitrogen mustard and aminopterin rapidly relieved tumors, they are both resistant, leading to disease recurrence [78, 79]. Various underlying mechanisms have been discovered for drug resistance development in the tumor, including tumor heterogeneity, some cellular level changes, genetic factors, and other novel mechanisms that have been highlighted in the past few years [80]. Overcoming drug resistance has become one of the most important goals in cancer research. Despite the large amount of research focusing on drug resistance in recent years, there is still a great lack of successful clinical approaches that can truly contribute to treatment. Recent studies have shown that iron-sulfur clusters play a role in part of the mechanism for drug resistance.

Cisplatin is a platinum-containing anticancer drug that has shown clinical efficacy against various solid tumors, including ovarian cancer, prostate cancer, malignant lymphoma, and squamous carcinoma of the head and neck. Iron-sulfur clusters are transferred from mitochondria to iron regulatory proteins (IRP), m-aconitases, and ferrochelatases by glutaredoxin 5 (GLRX5) [81]. Mutations in GLRX5 affect the production of downstream iron-sulfur clusters biosynthesis and maturation [82]. New studies have demonstrated that inhibiting GLRX5 induces ferroptosis in cisplatin-resistant head and neck cancer cells, suggesting a new therapeutic strategy for overcoming head and neck cancer chemoresistance through promoting ferroptosis by inhibiting GLRX5 [83].

There are strong similarities between the problem of drug resistance in cancer and that in infectious diseases, where highly proliferating intrinsic or extrinsic aggressors are ubiquitous [77]. Researchers found that during the respiratory burst in *Staphylococcus aureus* infections, ROS are generated that attack proteins containing iron-sulfur clusters, including TCA cycle enzymes, resulting in decreased respiration, decreased ATP, and increased antimicrobial resistance [84]. Therefore, the protection of iron-sulfur clusters may be able to counter antibiotic resistance.

As research on iron-sulfur clusters in drug resistance in cancer cells remains very limited, exploring the mechanisms of iron-sulfur clusters and their related proteins in drug resistance and their regulation may provide new directions for future research.

## 4. Iron-Sulfur Clusters and Regulated Cell Death

### 4.1. Iron-Sulfur Clusters and Ferroptosis

In 2012, Dixon et al. first described the concept of ferroptosis, a novel type of programmed regulated death elicited by the induction of cell death by the small molecules erastin and RSL-3 in Ras mutant cell lines [85]. Ferroptosis has been described as an iron-dependent form of regulated cell death (RCD) [86]. It is characterized as a form of cell death caused by cytoplasmic membrane damage or organelle membrane damage triggered by iron accumulation and membrane phospholipid peroxidation [87]. In terms of cell morphology, ferroptosis exhibits cellular morphological changes that differ from other types of RCD in that it does not exhibit either the cellular swelling observed in necroptosis and cell scorching or the cellular shrinkage and apoptotic vesicle formation exhibited in apoptosis. Ferroptosis also does not exhibit chromatin condensation in the nucleus or cytoskeletal disintegration in terms of organelle morphology; however, cellular mitochondria in ferroptosis exhibit significant changes, specifically mitochondrial disorders, including mitochondrial contraction, loss of mitochondrial cristae, and outer mitochondrial membrane (OMM) rupture [88].

Ferroptosis cell death was first discovered in the study of small-molecule inducers for cancer treatment, which provided a new idea for tumor treatment. Many studies have confirmed that ferroptosis *in vivo* and *in vitro* is effective against pancreatic cancer, breast cancer, nonsmall cell lung cancer, glioblastoma, and hepatocellular carcinoma cells [89–95]. More importantly, recent reports show that cancer cells resistant to conventional therapy or tend to metastasize are susceptible to ferroptosis and that the sensitivity of drug-resistant cells to chemotherapy and the effect of immunotherapy can be enhanced by ferroptosis [96–102].

Either extrinsic or intrinsic pathways can trigger ferroptosis. Broadly speaking, the extrinsic pathway is initiated by regulation of transporters (e.g., the cystine-glutamate reverse transport system Xc- or activation of the iron transporters transferrin and lactoferrin). In contrast, the intrinsic pathway is mainly induced by blocking the expression or activity of intracellular phospholipid peroxide-scavenging systems [e.g., glutathione peroxidase 4 (GPX4)], excessive phospholipid peroxidation, or imbalanced iron ion homeostasis. In general, induction of ferroptosis requires three hallmark conditions: (1) the presence of redox-active iron in the form of unstable iron pools and iron-dependent peroxidases such as lipoxygenase and cytochrome P450; (2) the presence of key substrates for peroxidation, i.e., phospholipids with polyunsaturated fatty acyl termini and diallyl carbons that are susceptible to peroxidation [e.g., the peroxidation of PUFAs by ALOXs or POR-mediated classical pathway [103] and peroxisome-ether-phospholipid axis-mediated nonclassical pathway [104]]; (3) dysregulation of complex lipid peroxidation repair networks, including glutathione-GPX4, GCH1 -BH4, and NADPH-FSP1-CoQ10 [105, 106], causing the mitochondrial dihydroorotate dehydrogenase (DHODH) pathways to be disrupted. Essentially, ferroptosis is caused by an imbalance in the dynamic balance between intracellular scavenging of peroxisomal phospholipids, lipid peroxidation, and iron accumulation, represented by GPX4.

A growing number of studies have identified that ferroptosis is metabolically regulated RCD. The iron-sulfur clusters, key group networks of iron metabolism and defenders of mitochondrial function, have received extensive attention in studies involving ferroptosis. Inhibition of iron-sulfur cluster synthesis causes activation of the iron starvation response. It inhibits iron-sulfur cluster synthesis, followed by an increase in lipid peroxidation and cell death markers after treatment with iron death-inducing drugs or oxidative stress-inducing compounds in most solid tumor cells [107]. Iron-sulfur cluster deficiency can regulate iron homeostasis and susceptibility to iron death through IRP2-dependent sensing [108]. Frataxin (FXN) was recently regarded as a key regulator of ferroptosis by regulating iron homeostasis and mitochondrial function. Inhibition of FXN expression impedes iron-sulfur clusters assembly, activates iron starvation stress, and significantly enhances erastin-induced lipid peroxidation by accelerating free iron accumulation, leading to severe mitochondrial morphological damage, including enhanced fragmentation and loss of cristae and subsequent cellular ferroptosis [109]. Overall, FXN, which is localized in the mitochondrial matrix and involved in the biosynthesis of iron-sulfur clusters, is a novel regulator of ferroptosis and a potential target for enhancing antitumor activity based on ferroptotic cell death.

Cancer cells produce CISD2 (nutrient deprivation autophagy factor-1; NAF-1), a metal-sulfur [2Fe-2S] homodimer protein that acts as a prognostic marker in a variety of cancers [110]. It is proposed that CISD2 promotes rapid cell proliferation by preventing mitochondrial unstable iron (mLI) and reactive oxygen species (mROS) overloads [111, 112]. In human breast cancer cells, disruption of CISD2 function leads to the accumulation of mitochondrial unstable iron (mLI), resulting in elevated mitochondrial ROS (mROS) levels and enhanced expression of tumor suppressor thioredoxin-interacting protein (TXNIP) associated with ferroptosis activation, which eventually causes ferroptotic cell death in tumor cells [113]. Furthermore, genetic inhibition of CISD1 (also termed mitoNEET) increases iron-mediated lipid peroxidation in mitochondria, which contributes to erastin-induced ferroptosis [114]. In addition, silencing GLRX5 activates the iron starvation response and increases intracellular free iron by increasing the binding of iron regulatory proteins (increased transferrin receptor and decreased ferritin) to iron-responsive elements. Inhibition of GLRX5 predisposes cisplatin-resistant head and neck cancer cells to ferroptosis [83].

Cysteine desulfurase (NFS1), a key biosynthetic protein, is an enzyme essential for iron-sulfur cluster biogenesis. Recently, a great deal of work has revolved around the functionality of NFS1, of which its inhibition regulates iron homeostasis and sensitivity to ferroptosis in cancer cells. Inhibition of NFS1 disrupts the iron-sulfur cluster biosynthesis, activates iron starvation responses, and triggers ferroptosis while inhibiting intracellular cysteine transport [107]. A genome-wide synthetic lethality-screening assay shows that targeting the CAIX-NFS1/xCT axis can regulate the vulnerability of solid hypoxic tumors [115].

A few applications of regulated iron-sulfur clusters could regulate cellular ferroptosis. Cysteine, an important substrate of the cellular thiophane-sulfur production system, produces persulfides as intracellular antioxidants and intermediates in iron-sulfur cluster production. Administration of plant thiane sulfide donors such as diallyl trisulfide (DATS) and dimethyl trisulfide (DMTS) prevented ferroptosis in osteosarcoma cells HT1080 treated with erastin, implying that ingestion of the trisulfide increased cellular resistance to ferroptosis [116]. Dihydroartemisinin (DHA) disrupts ISCU, thereby regulating iron metabolism, inhibiting mitochondrial function, suppressing GSH levels, promoting lipid peroxidation accumulation, and significantly inducing ferroptosis [52]. The stabilizing effect of pioglitazone on the iron-sulfur cluster of CISD1 inhibits mitochondrial iron uptake, lipid peroxidation, and subsequent ferroptosis [114].

The role of iron in ferroptosis is self-explanatory. For the iron-sulfur clusters can fuel ROS production [117], noncanonical ferroptosis induction is referred to ferroptosis that is initiated by increasing the LIP [118]. The LIP, which iron mostly in the form of Fe^2+^, can directly catalyze free radical formation through Fenton reactions, leading to the propagation of lipid peroxidation [119]. The classic chemodynamic therapy (CDT) is an antitumor therapy that directly employs endogenous chemical energy to trigger ROS burst and destroys cancer cells [120]. Specially, the mitochondria-targeting chemodynamic therapy nanodrugs (M-CDT nanodrugs) that can generate high levels of ROS at the mitochondrial site are proved to be spatial specificity and anticancer efficacy [120, 121]. It is found that a kind of nanoparticles effectively enhanced intracellular ROS level to activate ferroptosis pathway. And, the enhanced ROS induced the apoptosis pathway and decreased MMP-9 expression to synergize with ferroptosis for cancer therapy [122]. Also, a smart biomimetic metal organic framework based on ROS-ferroptosis-glycolysis regulation for enhanced tumor chemoimmunotherapy is reported [123]. Overall, the targeted regulation of iron-sulfur cluster biosynthesis to regulate cellular ferroptotic death, especially in tumor cells, represents a promising therapeutic strategy.

### 4.2. Iron-Sulfur Clusters and Cuproptosis

Copper, widely known to be essential for life, acts as a cofactor for vital enzyme activities [6]. However, intracellular copper concentrations are restrained at very low and moderate levels by active homeostatic mechanisms acting on a concentration gradient to prevent the accumulation of free intracellular copper that is harmful to cells [124]. When copper levels are too low, metal-binding enzymes become impaired, while when copper levels are too high, cells become overwhelmed and die [125]. In humans, the accumulation of excessive copper can be life-threatening, but selective killing of cancer cells can be achieved using a more concentrated increase in intracellular copper [126]. Emerging studies have shown that Copper ionophores, the small molecular tools that bind copper, can shuttle copper into cells [127]. Numerous lines of evidence indicate that copper ionophores carry copper ions to induce cell death by intracellular copper ion accumulation [127, 128]. Recently, Tsvetkov et al. revealed that copper toxicity involves the disruption of specific mitochondrial metabolic enzymes, triggering an unusual mechanism of cell death, which could explain the pathology associated with inherited copper overload diseases and suggest new ways to use copper toxicity to treat cancer [129]. This copper-dependent cell death was named cuproptosis. Recent studies have shown that cuproptosis is mediated by the lipoylation protein [129], with impaired mitochondrial metabolism integral to cuproptosis [129, 130]. It is well known that very few mammalian proteins can be lipoylated and concentrated in the TCA cycle, where lipoylation is required for enzymatic function and is essential for mitochondrial metabolism [131, 132], hence the need to understand the relationship between mitochondrial activity and susceptibility to cuproptosis. Tsvetkov et al. revealed the features of cuproptosis, which include (1) increased levels of lipid acylated TCA enzymes (especially PDH complexes) in cells with active respiratory TCA cycles, with the lipid acyl fraction acting as a direct copper binder, leading to aggregation of lipid acylated proteins, loss of iron-sulfur clusters-containing proteins; and (2) induction of HSP70, reflecting acute proteotoxic stress and ultimately cell death [129]. Tsvetkov et al. confirmed that FDX1 (which encodes a reductase known to reduce Cu^2+^ to its more toxic form, Cu^1+^, and to be a direct target of elesclomol) and protein lipoylation are the key regulators of copper ionophore–induced cell death [129].

Specifically, the occurrence of cuproptosis is controlled by several regulators/mechanisms [129, 130, 133]. (1) FDX1 and six genes encoding components of the lipoic acid pathway including [lipolytransferase 1 (LIPT1), lipoyl synthase (LIAS), and dihydrolipoamide dehydrogenase (DLD)] or protein targets of lipoylation [the pyruvate dehydrogenase (PDH) complex, including dihydrolipoamide S-acetyltransferase (DLAT), pyruvate dehydrogenase E1 subunit alpha 1 (PDHA1), and pyruvate dehydrogenase E1 subunit beta (PDHB)] were identified as key genes that promote copper death. (2) The copper binding to lipoylated TCA cycle proteins resulted in lipoylation-dependent oligomerization of DLAT, and copper directly binds and induces the oligomerization of lipoylated DLAT, whereby FDX1 and protein lipoylation are upstream regulators of this process. (3) Copper delivered to mitochondria by copper ion carriers binds directly to these lipidated proteins, forcing them to form long protein chains and clusters that lead to cell death. (4) Copper can destabilize iron-sulfur clusters–containing proteins, which is part of several key metabolic enzymes. This causes these metabolic enzymes to downregulate their expression, putting the cells into a toxic state of stress that ultimately kills them. The fact that actively respiring cells are susceptible to cuprotosis seems consistent with a Copper ionophore-elesclomol, which is more clinically effective for mitochondrial-dependent cancer cells. The cuprotosis susceptibility of cells in a more respiratory state may be because more lipid acylases will be expressed, resulting in more aggregates. Furthermore, higher metabolic flux through lipid acylases, such as DLAT, may increase their affinity for copper, resulting in greater aggregation during active respiration.

Although few research studies examine cuproptosis with cancer treatment, copper and copper ionophores therapy have shown a unique fascination in cancer treatment. Copper status can serve as a vulnerability for cancer. The two main therapeutic approaches currently targeting this nutrient include Cu(I) chelators to deplete copper pools, thereby driving tumor proliferation and metastatic pathways or copper ion carriers to replenish copper and cuprotosis [134]. Several ways of copper supplementation to cancer cells have potentially important value in cancer drug-resistance therapy and clinical treatment. Initially approved by the FDA in 1951, disulfiram inhibits aldehyde dehydrogenase (ALDH) to treat alcohol dependence [135], and subsequent studies have shown the potential of disulfiram in combination with copper ions (Cu(II)) to treat a variety of human cancers [136]. Clinical trials have confirmed the anticancer and/or chemosensitizing effects of DSF or Cu-DSF, particularly in glioblastoma [137]. Another copper-binding compound, elesclomol, was initially screened by a cell-based phenotypic screen for small molecules that could enhance the antitumor activity of paclitaxel [138]. Elesclomol, in combination with paclitaxel, has been used with some success in many clinical trials, mainly for advanced melanoma [139, 140]. A 3-phase combination clinical trial in chemotherapy-naïve patients with advanced melanoma showed a lack of efficacy with the combination of erexicolol and paclitaxel, but post hoc analysis showed antitumor activity in patients with low plasma lactate dehydrogenase (LDH) levels [141]. Low LDH reflects higher dependence of cells on mitochondrial metabolism, which is consistent with the action of elesclomol requiring active mitochondrial respiration. Overall, tumors that depend on mitochondrial metabolism may be particularly sensitive to cuproptosis [130, 142].

Elesclomol is an anticancer drug that targets mitochondrial metabolism and inhibit cancer by inducing cuproptosis. Cancer cells that are heavily dependent on mitochondrial metabolism are extremely sensitive to elesclomol, and existing studies have reported significant inhibition of elesclomol in a variety of cancer cells, including cancer stem cells, drug-resistant cells, and cells with low glycolytic activity. There are many clinical trial data demonstrating the safety of eletriptol in clinical applications [139–141]. Iron-sulfur cluster proteins play a key role in cuproptosis and their homeostasis is involved in cuproptosis-dependent cancer therapy. Several recent studies have shown that cuproptosis-associated genes, including iron-sulfur clusters, are potential predictors of diagnostic, prognostic, and therapeutic response in some cancers and are associated with tumor microenvironment, immune response and immunotherapy [143–147].

### 4.3. Iron-Sulfur Clusters and PANoptosis

Programmed cell death plays a crucial role in organismal development and host defense. Apoptosis, necroptosis, and pyroptosis are the most well-known types of programmed cell death [148]. Inflammatory disorders, cancer, and other pathological conditions are linked to these proteins, which are involved in cell damage, transformation, and elimination of infected cells. However, mounting evidence indicates significant crosstalk between the three pathways [149]. As a synergistic pathway that covers pyroptosis, apoptosis, and necroptosis, PANoptosis is initiated by certain triggers and controlled by PANoptosome complex [150]. PANoptosis induces inflammatory cell death by triggering pyroptosis, apoptosis, and necroptosis collectively allowing pathogens to inhibit individual cell death. There are many diseases associated with PANoptosis, including autoinflammatory diseases, neurodegenerative diseases, cancer, microbial infections, and metabolic diseases [151].. It is proven that regulating caspase-8 in PANoptosis provided new strategies and targets for cancer [152]. A novel study has reported that interferon regulatory factor 1 (IRF1) regulates PANoptosis to prevent colorectal cancer [14]. Under certain circumstances, Z-DNA-binding protein 1 (ZBP1) is an important mediator of NLRP3 inflammasome activation and PANoptosis. As part of PANoptosis and PANoptosome assembly during influenza virus infection, ZBP1-NLRP3 inflammasomes are formed [153]. It is reported that adenosine deaminase acting on RNA 1 (ADAR1) restricts ZBP1-mediated immune response and PANoptosis to promote tumorigenesis, which reveals that PANoptosis is a potential target for cancer treatment [154]. As molecular switching from one form of cell death to another could be an effective strategy for efficiently killing cancerous cells, PANoptosis could be an effective target in enhancing the effectiveness of cancer therapy.

Iron-sulfur cluster biogenesis is regulated by cysteine desulfurase (NFS1), an enzyme that plays a crucial role in iron-sulfur cluster assembly and several iron-sulfur cluster-dependent pathways [107]. Under oxaliplatin treatment, NFS1 inhibits PANoptosis in a S293 phosphorylation-dependent manner, suggesting that iron-sulfur cluster regulation may be a novel regulatory strategy of PANoptosis in cancer. Triggering PANoptosis by regulating the iron-sulfur cluster can also provide a promising strategy for improving the outcome of platinum-based chemotherapy in the treatment of cancer. However, there are only a few reports on the mechanism of iron sulfur cluster in PANoptosis and its relationship with cancer. The PANoptosis caused by biogenesis of iron sulfur cluster remains to be studied.

### 4.4. Iron-Sulfur Clusters and Apoptosis

In 1972, Kerr et al. created the term apoptosis (a-po-toe-sis) to describe a morphologically distinct form of cell death [155]. Apoptosis is a form of programmed cell death that results in the orderly and efficient removal of damaged cells. The process of apoptosis plays an important role in a variety of processes, including cell turnover, immune system development, hormone-dependent atrophy, embryonic development, and chemical-induced cell death [156]. Cancer is characterized by deregulation of the apoptotic cell death machinery [157]. The caspase is both the initiator and the executor of apoptosis. There are three. It is possible to activate caspases in two ways: intrinsically (or mitochondrial) and extrinsically (or death receptor), both leading to a common pathway or execution phase [158]. These underlying apoptosis mechanisms play a crucial role in the pathogenesis of many diseases. As cancer advances, its apoptotic cell death machinery deregulation becomes more pronounced [157]. Apoptosis is a popular target of many cancer treatment strategies. Research suggests it is possible to target apoptosis in cancer [158].

The mitochondrial electron transport chain utilizes a series of electron transfer reactions to generate cellular ATP through oxidative phosphorylation. The electron transfer can cause generation of ROS, leading to homeostatic signaling and oxidative stress during pathology [159]. Therefore, iron-sulfur clusters are an important part of the mitochondrial electron transport chain and are tightly linked to ROS production. In aerobic eukaryotes, mitochondrial respiration generates most of the energy. Through four protein complexes in the mitochondria inner membrane, NADH and FADH2 donate electrons to the last electron acceptor, oxygen, in oxidative phosphorylation. It is found that iron-sulfur clusters are essential to the function of most respiratory complex proteins, and mutations in genes encoding proteins required for biogenesis of iron-sulfur proteins result in reduced activity of the respiratory chain [160]. Electrons can be transferred directly by reducing Fe^3+^ to Fe^2+^ in cytochromes and iron-sulfur proteins [161]. Multi-iron sulfur clusters are found in respiratory complex I (NADH) and II (succinate) that establish electron-tunneling chains one at a time to ubiquinone (Q). Complex III contains only a single iron-sulfur protein. Iron-sulfur proteins play multiple roles in mitochondrial iron homeostasis and apoptosis. Iron and ROS are regulated by mitoNEET, a protein in the outer mitochondrial membrane [162]. It contains a redox-active [2Fe-2S] cluster [163]. Induction of cell death by TNF*α* is mediated by binding of Stat3-Grim-19 to mitoNEET, which forces the release of the iron-sulfur cluster. As a result, mitochondria accumulate iron in the matrix, causing the elevated levels of ROS and mitochondrial damage, leading to apoptosis [164]. Furthermore, cancer cells can transfer their [2Fe-2S] clusters to apolipoprotein receptors via mitoNEET, which has been linked to proliferation [165]. This confirms the role of iron-sulfur cluster-associated apoptosis in cancer. Nanomedicine can improve the solubility and absorption rate of poorly soluble drugs compared with traditional drugs, as well as reduce adverse reactions, improve targeting, and accurate control release. The ROS-based nanomaterials are reported to have excellently therapeutic effects for myocardial infarction reperfusion [166]. Therefore, decreasing ROS levels through nanomaterials may be an important approach to treat cancer by modulating iron-sulfur cluster-associated apoptosis.

**Figure 1:**
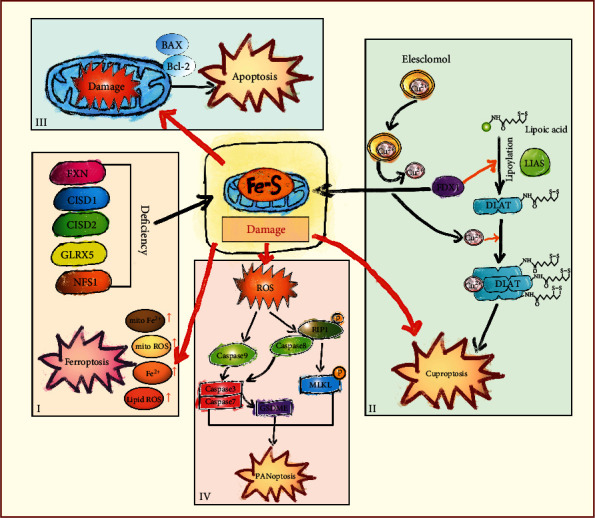
The schematic diagram of the link between iron-sulfur clusters and multiple regulated cell death. (i) The deficiency of several proteins involved in iron-sulfur clusters biosynthesis and homeostasis including FXN, CISD1, CISD2, GLRC5, and NFS1 causes iron-sulfur clusters damage, resulting in an imbalance in mitochondrial homeostasis and a rise in mito ROS, mito Fe^2+^, intracellular Fe^2+^, and lipid peroxidation eventually causing ferroptosis. (ii) The loss of iron-sulfur clusters proteins triggered proteotoxic stress and cell death in cuproptosis. Copper directly binds to lipoylated components of the tricarboxylic acid (TCA) cycle, then the aggregation of these copper-bound, lipoylated mitochondrial proteins and subsequent iron-sulfur clusters protein loss triggered cuproptosis. (iii) The homeostatic imbalance of iron-sulfur clusters causes mitochondrial damage, abnormal accumulation of ROS and the regulation of apoptotic regulatory proteins BAX and Bcl-2, which in turn cause the onset of apoptosis. (IV) The phosphorylated NFS1 is involved in PANoptosome assembly and PANoptosis by regulating the biosynthesis of iron-sulfur clusters and thus the level of ROS.

The cytosolic iron-sulfur protein assembly (CIA) mechanism that assembles iron-sulfur clusters in the cytoplasm is also involved in apoptosis. Cytokine-induced apoptosis inhibitor 1 (CIAPIN1), one of the anamorsin protein family, is a newly identified cytokine-induced apoptosis inhibitor, which has been shown to function as an antiapoptotic protein by regulating Bcl-2 and Bax [167]. Anamorsin carries a [2F-2S] cluster that is part of the electron transport chain early in the CIA pathway [168]. Dre2, the yeast homolog of anamorsin, is also an iron-sulfur protein that receives electrons from Tah18 during CIA [169]. Yeast cells exposed to lethal doses of H_2_O_2_ lose Tah18-Dre2 interaction, and Tah18 relocalizes to mitochondria. Apoptosis is promoted and outer membrane integrity is compromised [170]. More importantly, in addition to its prognostic value for human tumors and involvement in cancer progression, CIAPIN1 is also proposed as a potential target for new anticancer interventions [171]. In summary, iron-sulfur clusters in mitochondria play an integral role in apoptosis, and these mechanisms of action are relevant to cancer targets (Figure 1).

## 5. Conclusions

Iron-sulfur clusters, an ancient and conservative regulator of life, seems to be shedding new light with the rise of numerous novel research fields. Programmed death has been a major concern in cancer therapy, and with the expansion of research horizons, the relationship between iron-sulfur clusters and the multiple programmed deaths has entered the public eye. As an important iron homeostatic link in cells, the relationship between iron-sulfur clusters and ferroptosis is clear. And in the mitochondria-dependent modulation program of cuproptosis, iron-sulfur clusters are a key element. Moreover, as an important modulator of the mitochondrial microenvironment, the role of iron-sulfur clusters in apoptosis and PANoptosis seems to be “unquestionable.” However, there are still many questions about the details of iron-sulfur clusters in the modulation of multiple cell death since emerging programmed death modalities such as ferroptosis and cuproptosis have been studied for a short period of time. Iron-sulfur clusters act as a key player in several cell death modalities, but which cellular programmed death modality is preferentially induced by therapies targeting iron-sulfur clusters and if there is a relationship between time, dose, and means in the type of cell death induced, these are still questions to be explored. Although, there are relevant studies that have recently explored the link between ferroptosis and cuproptosis, as well as studies that have explored the relevance and timing of several cell death modalities, the role that iron-sulfur clusters play in this is unclear. In addition, it is still not clear whether iron-sulfur clusters are associated with the onset of cell death type with respect to cell type. On the other hand, it is also of interest whether iron-sulfur clusters are again involved in regulating several cell death modalities through regulation of the microenvironment. Interventions targeting iron-sulfur clusters in programmed cancer death are also in need of further development.

There remain many questions about the role of iron-sulfur clusters in health and disease, and there still many studies needed from basic biochemistry and translational science perspectives to find therapeutic strategies, and the importance of targeting iron-sulfur clusters in tumor cells, but in general, targeting iron-sulfur clusters biogenesis offers potential opportunities for inhibiting cancer development, drug resistance, and developing cancer therapy. It is also considered that the development of drugs for iron-sulfur clusters as adjuvants or combinations of existing therapeutic drugs in cancer therapy. For example, the drugs targeting iron-sulfur clusters are used as adjuvants for sensitization and antiresistance in combination with existing chemotherapeutic drugs. The efficacy of treatments targeting iron-sulfur clusters in cancer is still controversial, while several iron-sulfur clusters have been described as “repairable” and iron-sulfur clusters biosynthesis may only be temporarily impaired. In addition, the development of targeted drugs for iron-sulfur clusters should also focus on the impact on normal cells, and the long-standing strategy of developing drugs for metabolic differences between cancer and normal cells may be equally applicable in this context. Indeed, inducers of ferroptosis and inducers of cuproptosis have been found to be effective in cancer cells at doses that have no effect on normal cells. Therefore, given the role of iron-sulfur clusters in multiple programmed deaths, drug development strategies targeting iron-sulfur clusters can be based on the sensitivity of cells to programmed death for the establishment of relevant systems. Some studies have shown that iron-sulfur clusters-targeting drugs may not always lead to rapid cell death. Therefore, therapies that target iron-sulfur clusters in combination with several modalities of regulatory-programmed death induction are potentially valuable for developing cancer treatment, mitigating drug resistance, and enhancing existing therapies. Given the critical importance of iron-sulfur clusters in multiple RCDs, future investigation into the effects of iron-sulfur clusters in multiple RCDs, combined therapeutic strategies between iron-sulfur clusters and RCD induction, and potential links between iron-sulfur clusters and RCD induction in cancer cells resistance may uncover new therapeutic avenues.

All in all, researchers have elucidated a number of regulatory mechanisms and signaling pathways of iron-sulfur clusters, and iron-sulfur clusters biosynthetic pathways and homeostasis seem to represent a critical vulnerability in a class of cancer cells. It has been demonstrated that iron-sulfur clusters are closely associated with a variety of programmed cell death in cancer therapy, and therefore the rational use of these mechanisms in the biomedical field to address iron-sulfur clusters as a target for drug development and therapeutic strategies could be a promising option.
